# Commentary: Analysis of case management service quality for PCI patients based on the SERVQUAL model: a single-center prospective study

**DOI:** 10.3389/fpubh.2026.1790482

**Published:** 2026-03-30

**Authors:** José A. Martínez, Laura Martínez

**Affiliations:** Department of Business Economics, Technical University of Cartagena - Member of European University of Technology, Cartagena, Spain

**Keywords:** importance-performance analysis, percutaneous coronary intervention, satisfaction, service quality measurement, SERVQUAL model

## 1 Introduction

In their article “Analysis of case management service quality for PCI patients based on the SERVQUAL model: a single-center prospective study,” Wang et al. (1) analyze the quality of case management in patients undergoing percutaneous coronary intervention (PCI) using the SERVQUAL (SERVice QUALity) model and importance-performance analysis (IPA). Although the topic is highly relevant for hospital management, the methodology employed contains conceptual and statistical problems that have been superseded in the specialized literature. The reliance on predefined dimensions, the redundancy of expectations, the imprecision in sampling, and particularly the fundamental misapplication of the IPA methodology significantly undermine the validity of the findings.

## 2 The imposition of dimensions vs. patient voice

Wang et al.'s (1) primary weakness is the use of a structured questionnaire based on SERVQUAL's 22 generic dimensions, which imposes the researcher's perspective over the patients. Recent research in cardiology services demonstrates that the use of associative maps and semantic priming techniques allows the identification of which attributes patients genuinely associate with satisfaction in a free and spontaneous manner (2). The study by Consuegra-Sánchez et al. (2) has shown that professional and personal care is the dominant attribute (mentioned by 97% of patients), while other factors such as food or waiting times have significantly different weights than those assumed in generic scales. In addition, forcing patients to evaluate dimensions such as tangibility through the personal appearance of managers may generate artificial results, as overall service quality is a complex and multifaceted construct that does not always conform to the rigid SERVQUAL framework.

## 3 The redundancy of the expectations paradigm

Wang et al. (1) calculate quality as the difference between perceptions and expectations (P–E), a methodologically redundant practice. It has been demonstrated that when a patient evaluates service performance, they are already making an implicit comparison with prior expectations in their subjective judgment (3).

Measures based exclusively on perception (such as SERVPERF - SERVice PERFormance model-) are psychometrically superior in construct validity and operational efficiency. Additionally, the use of difference scores introduces reliability problems, as expectations typically show low variability and positive bias, converting the “negative gap” into a statistical artifact of the measurement method rather than a reflection of an actual service shortcoming (4).

## 4 The problem of multidimensionality and the “chord” metaphor

The analyzed article assumes that the overall quality is a linear sum of attributes. However, the overall quality should be understood as a complex phenomenon, akin to a musical chord (5). While individual attributes are the “notes,” the overall quality is the resulting “chord,” whose perception is qualitatively distinct from the sum of its parts (3).

Attempting to deconstruct quality into predefined dimensions ignores the fact that the whole is more than the sum of its notes. Moreover, halo effects contaminate the evaluation of specific attributes once the patient has formed an overall judgment. This reduces the diagnostic value of measuring 22 separate items. A more robust approach would be to isolate the global perception of quality (typically assessed with a single item) from the evaluation of specific operational attributes.

## 5 Imprecision in sample size and interval scales

Wang et al. (1) calculate their sample size based on the variance of expectations but ignore the intrinsic imprecision of the measurement scale. It is a common error to confuse the variance of interval scales with that of binomial proportions or to overlook how the scale range (1–5 or 1 to 5) affects estimation precision (6). The use of the Measurement Scale Imprecision Factor (MSIF), an index invariant to scale range, is recommended to allow a robust comparison of imprecision across different studies with Likert and other interval scales of different lengths, which is common in the service quality literature; for example, the creators of the SERVQUAL model used a seven-point scale (7).

## 6 Statistical deficiencies in IPA implementation and methodological misuse

The IPA analysis presented in Wang et al.'s (1) study contains a fundamental methodological error that deserves particular attention. Although the authors cite Martilla and James (8), the originators of the IPA technique, they misapply the method's core logic. In the original IPA framework, the horizontal axis should represent attribute importance, not expectations, while the vertical axis represents actual performance. The distinction is critical: importance reflects how much a feature matters to consumers, whereas expectations reflect what consumers anticipate they will receive.

Wang et al. (1) confound these concepts by using expectation scores on the horizontal axis as a proxy for importance. This conflation, however, fundamentally distorts the interpretation of the IPA. Expectation levels and importance ratings are conceptually and empirically distinct constructs. A patient may have low expectations for rapid service completion yet consider it highly important; conversely, patients may expect extensive personalized care without attaching high importance to it. By substituting expectations for importance, the authors create a hybrid measure that obscures the true structure of patient preferences and misguides quality improvement priorities.

Furthermore, the graphical representation lacks horizontal and vertical error bars. A graph showing only mean points is misleading, as it cannot determine whether an attribute's location in a “priority for improvement” quadrant is statistically significant or whether uncertainty causes overlap between quadrants due to variance. When working with a difference function (P–E), the authors neglect error propagation. The error in the mean of perceptions and that of expectations combine, increasing uncertainty in the IPA diagram's point positions (9). Failing to account for this propagation using methods such as Taylor series approximations means that any management recommendations derived from the graph lack statistical rigor.

## 7 Discussion

Although the study by Wang et al. (1) aims to improve case management in PCI, their reliance on outdated models such as SERVQUAL, coupled with a fundamentally flawed IPA implementation, severely undermines the practical utility and validity of their conclusions. Future research should prioritize the patient's voice through associative maps, employ direct perception measures, and include error bars and uncertainty propagation in any importance-performance analysis, remaining faithful to the original IPA methodology as established by its creators.
